# Counting Cats: The integration of expert and citizen science data for unbiased inference of population abundance

**DOI:** 10.1002/ece3.7330

**Published:** 2021-04-02

**Authors:** Jenni L. McDonald, Dave Hodgson

**Affiliations:** ^1^ Veterinary Department, Cats Protection National Cat Centre Haywards Heath UK; ^2^ Bristol Veterinary School University of Bristol Bristol UK; ^3^ Centre for Ecology and Conservation College of Life and Environmental Sciences University of Exeter Penryn UK

**Keywords:** abundance, citizen science, false positive, integrated abundance model, integrated model, misidentification, population size

## Abstract

Free‐roaming animal populations are hard to count, and professional experts are a limited resource. There is vast untapped potential in the data collected by nonprofessional scientists who volunteer their time to population monitoring, but citizen science (CS) raises concerns around data quality and biases. A particular concern in abundance modeling is the presence of false positives that can occur due to misidentification of nontarget species. Here, we introduce Integrated Abundance Models (IAMs) that integrate citizen and expert data to allow robust inference of population abundance meanwhile accounting for biases caused by misidentification. We used simulation experiments to confirm that IAMs successfully remove the inflation of abundance estimates caused by false‐positive detections and can provide accurate estimates of both bias and abundance. We illustrate the approach with a case study on unowned domestic cats, which are commonly confused with owned, and infer their abundance by analyzing a combination of CS data and expert data. Our case study finds that relying on CS data alone, either through simple summation or via traditional modeling approaches, can vastly inflate abundance estimates. IAMs provide an adaptable framework, increasing the opportunity for further development of the approach, tailoring to specific systems and robust use of CS data.

## Graphical abstract

Integrated Abundance Models (IAMs) address the key challenge in abundance modelling of false positives that can occur due to misidentification of nontarget species. Integrating data sources of differing quality improves precision of abundance estimates by accounting for misidentification biases. The framework described is adaptable, and we hope it provides a useful introduction to the concept to allow further optimization of the approach and tailoring to specific systems

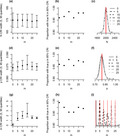

## 1 INTRODUCTION

Monitoring of animal populations relies increasingly on data collected by the public (e.g., Dickinson et al., 2012; Theobald et al., 2015). This dependency on citizen science (CS) is only likely to increase further, with the development of more sophisticated open‐access web applications (Silvertown, 2009), smartphone technology (Kim et al., 2013; Liebenberg et al., 2017; Rowley et al., 2019; Teacher et al., 2013) and crowdsourcing for data, alongside the traditional long‐term CS studies that historically have relied on public input (e.g., Dennis et al., 2017; Newson et al., 2012; Sullivan et al., 2014). Ideally, study design should attempt to minimize biases (Altwegg & Nichols, 2019); however, this is not always possible especially when trying to make robust inference from opportunistic, historic and crowd‐sourced data collection. Model‐based approaches offer an alternative, pragmatic, cost‐effective solution to improve accuracy and account for uncertainty in parameter estimates (Van Strien et al., 2013).

Wildlife abundance is of central interest in many studies, as its inference is required to assess the status of a population to inform conservation, welfare, and management goals. However, abundance estimates are functions of detection probability, whereby reported counts are unlikely to be a true estimate of population size. It is largely accepted incomplete detection can bias inference. Indeed, n‐mixture models (also termed binomial‐mixture models) (Royle, 2004) are commonly used to correct survey data for false negatives and provide an adjusted measure of abundance. Although n‐mixture models are often more effective than using raw counts alone (Kidwai et al., 2019), they rely on a key assumption that false positives do not occur. But, false positives can occur due to misidentification (Hull et al., 2010; Molinari‐Jobin et al., 2012; Shea et al., 2011; Tillett et al., 2012), where nontarget species or subgroups are incorrectly identified and counted as a target individual. Misidentification can also occur with environmental DNA, that may be more prone to false positives due to sample contamination (Guillera‐Arroita et al., 2017) and is relevant to sign surveys, such as scat from the target species (Janečka et al., 2008), where nontarget species can be incorrectly incorporated in abundance estimates. When false positives occur, models that only account for false‐negative errors will yield inflated estimates of abundance (Link et al., 2018). Tackling this problem requires data integration that accounts for data sources being observed with error.

Model‐based integration of data sets is not new to ecology. Indeed, it is advocated and used to model species distributions (Isaac et al., 2020), demographic processes (Schaub & Abadi, 2011), and occupancy (Ruiz‐Gutierrez et al., 2016) whereby the integration process allows ecologists to combine data sets while retaining their relative strengths. Although there are potentially many forms of data integration (Fletcher et al., 2019), here we focus on formal statistical integration, which takes into account the unique biases of each data set. Such an approach has been found to account for spatial biases to improve predictive performance and accuracy in distribution models (Dorazio, 2014; Fithian et al., 2015; Fletcher et al., 2019) and to improve our understanding of demographic processes in integrated population models (Abadi et al., 2010; McDonald et al., 2016; Weegman et al., 2016). The development of data integration in an abundance framework provides opportunities to similarly make best use of data sources.

Here, we focus on integrating a high‐quality and low‐quality data set, derived independently from expert and CS collection processes, respectively. Specifically, the inclusion of a subset of high‐quality abundance data permits some sites to be assigned with a greater degree of certainty. The type of high‐quality data required will vary as a function of several factors, including the surveyor's expertise, geographic area, and the species involved. Expert surveys could come from any approach with high detectability, such as intensive surveillance (Mills et al., 2016), aerial surveys such as with large animals, or indirect verification such as through images (Gardiner et al., 2012; Lye et al., 2012) or acoustic telemetry (Vianna et al., 2014).

In the following sections, we describe misidentification as a source of false‐positive observations and present an abundance model that borrows inference from high‐quality data to estimate misidentification of nontarget individuals and consequently improve all estimates of abundance. Due to the integration of data sources, we term this an integrated abundance model (IAM). We test model performance under different scenarios, including the degree of variation in the high‐quality data, the prevalence of high‐quality data included, bias in detection data and ecological differences.

To demonstrate the approach further, we describe a case study of our own application of an IAM to estimate the number of unowned cats within an urban area. Determining the number of unowned cats in urban areas is difficult in part because of problems accurately distinguishing owned from unowned cats. Indeed, many research studies in urban areas focus on free‐ranging cat populations due to an inability to differentiate between cat subgroups (Elizondo & Loss, 2016; Flockhart et al., 2016; Hand, 2019). A further problem, in these urban areas, is the inability of researchers to access private locations such as those behind residential properties and businesses (Hand, 2019; Kilgour et al., 2017). Citizen science approaches have the potential to provide information on abundance from otherwise inaccessible locations; however, the key difficulty of accurate differentiation between owned and unowned cats remains. An IAM approach offers a solution by integrating CS data with expert data that apply robust protocols to ensure accurate identification of an unowned cat.

Our simulation analyses and case study demonstrate potential for IAMs to provide robust and unbiased inference of abundance, which we hope will help to promote this issue further and enable further model development in species abundance studies.

## 2 METHODS

### 2.1 A brief outline of traditional abundance models

N‐mixture models are described briefly here for context but are explained extensively elsewhere (Kery & Royle, 2010; Royle, 2004). They can estimate species abundance from count data by accounting for imperfect detection, wherein not all individuals are seen. Unlike classical capture–mark–recapture approaches, N‐mixture models do not require the identification of individuals and instead depend on data from survey counts that are replicated in space. In short, they model two processes simultaneously:

1. Ecological. The species has a local abundance in the *i*th site (*N_i_*) with spatial variation at each site described by a Poisson distribution with a mean (*λ_E_*).

$$N_{i}∼\text{Poisson}\left(\lambda_{E}\right)$$

- Observation. The observed counts at each site and during each replicate survey (*j*) are described by a binomial distribution with a sample size *N_i_* and detection probability *p*.

$$y_{i,j}|N_{i}∼\text{Binomial}\left(N_{i},p\right)$$

Thus, inputs to the model are the replicate counts, which then yield estimates of detection probability and abundance. In these models, detection probability relates to incomplete detections only; hence, inference relies on the assumption that false positives do not occur. When counts risk the inclusion of misidentification of nontarget species, inferences will be biased ((Link et al., 2018); Appendix S2; Figure S1).

### 2.2 False positives

In the context of integrated abundance modeling, we define false positives as misidentification, whereby nontarget individuals are wrongly identified as the target individual. We recognize that false positives can also be a function of overdetection defined at the level of the individual, in other words the probability of multiple counts per individual, or overcounting. Bias due to overcounting is not discussed here, but could be explored in a future development of an IAM.

### 2.3 Overview of IAM

Existing Bayesian approaches to N‐mixture models provide the basic framework to model abundance from replicate counts. Here, we propose an integrated modeling approach to analyze multiple data sets simultaneously. Specifically, an IAM differs from an N‐mixture model in two key ways: (a) The addition of a independently collected high‐quality data set (*w_i_*), whereby expert consensus is available on the abundance of individuals in some, but not necessarily all sites; (b) an observation process that can account for both false‐positive and false‐negative errors in the observed replicate counts for each site (*y_i,_*_j_). IAMs assume that replicate counts are conducted over a period of population closure.

### 2.4 Expert data

IAMs account for observation error in expert counts.

$$w_{i}∼\text{Poisson}\left(N_{i}\right)$$

whereby observed expert counts (*w*) at sites *i* are linked to true site‐specific population sizes (*N_i_*) via a Poisson distribution, which is suited due to its natural constraints to yield integer values of zero and above. Such assumptions are commonly seen in integrated population models, specifically the modeling of population count data, whereby counts through time are assumed to not be subject to systematic biases, but rather observation error (Kéry & Schaub, 2011; Schaub & Abadi, 2011).

We additionally assume that where expert counts are available they are accurate at the level of presence or absence. This assumption is already implicit when using a Poisson distribution for observation error as if expert counts are zero, variance is also zero. However, to retain flexibility in the modeling approach under different observation errors, such as a normal distribution, and to allow explicit calculation of occupancy we include an additional binary layer of true occurrence.

$$z_{i}∼\text{Bernoulli}\left(\Omega \right)$$

$$N_{i}=z_{i}\lambda_{i}$$

whereby *z_i_* is a binary measure of occurrence, with each of the *i* sites occupied or not, that is modeled as a Bernoulli random variable determined by occupancy probability (Ω). True site‐specific population sizes (*N_i_*) are therefore a function of whether a site is occupied or not and a site‐specific mean *λ_i_*. When expert data on occurrence can be inferred from expert consensus, this can be included in *z_i_*.

A feature of the Poisson distribution is that its variance is equal to its mean. Although this assumption is commonplace in many demographic studies (Abadi et al., 2010; McDonald et al., 2016; Weegman et al., 2016), it may not be valid for some expert collected data. Indeed, if data were more or less variable than that modeled by a Poisson distribution, we would unnecessarily understate or overstate uncertainty in expert precision. While integer‐based distributions are most appropriate for count data, to highlight the adaptability of this approach we also provide an alternative option whereby a scaling parameter (*k*) can be included to mimic overdispersion or underdispersion of the observation error variance.

$$w_{i}∼\text{Normal}\left(N_{i},kN_{i}\right)$$

Here, observed expert counts (*w*) at sites *i* are linked to true site‐specific population sizes via a mean of *N_i_* and a variance scaled to *N_i_*. A *k* less than one would imply reduced variance relative to a Poisson distribution and *k* greater than one implies increased variance relative to Poisson. The above enables variance to be zero (or in practice specified to be relatively small for computational purposes) if a site is unoccupied. An additional option would be to obtain estimates of experts’ precision, through the collection of supplementary data, and use these estimates as prior information in the IAM.

Similar to observation models in other frameworks (Kéry & Schaub, 2011), we find within our simulations the choice of Poisson of Normal error structure does not introduce systematic biases (Appendix S2); therefore, we present our simulations in the main manuscript using the Poisson observation error, which is more appropriate for count data. However, bias derived from assumed distributions of observation error should be considered and alternative distributions, such as negative binomial or zero‐inflated Poisson may be equally or more appropriate depending on the study system.

### 2.5 Citizen science data

The CS data consists of spatial and temporal replicates. Instead of applying an N‐mixture model, an IAM accounts for both detection probability and misidentification of target species in CS counts.

$$y_{i,j}∼\text{Poisson}\left(N_{i}p+m\right)$$

whereby observed CS counts (*y_i,j_*) at each site *i* and during each replicate survey *j* are linked to true site‐specific population sizes (*N_i_*) via a detection probability (*p*) and the expected number of misidentifications (*m*). We apply a Poisson distribution to account for additional stochasticity in spatial replicates not accounted for in the systematic biases (*m* and *p*).

### 2.6 Joint likelihood

Inference is based on the joint likelihood that is a product of the likelihoods from the single data sets. Expert data are not required for all sites, but there must be some overlap of expert data and CS data for a subset of sites. A key element is that N_i_ is a common parameter to both the CS data and expert data. Combining their likelihoods borrows strength from the few well‐studied sites to infer detection biases across locations and to inform abundance estimates. A benefit of this particular integrated modeling approach is that it allows estimation of misidentification, which is otherwise unidentifiable from CS data alone.

$$L_{\text{IAM}}\left(y_{i,j},w_{i},z_{i}|N_{i},\Omega ,p,m\right)=L_{\text{CS}}\left(y_{i,j}|N_{i},p,m\right)XL_{E}\left(w_{i},z_{i}|N_{i},\Omega \right)$$

### 2.7 Simulation study

To test the performance of the IAM, we simulated pseudo‐data using known parameter values to create 100 independent data sets for 30 different scenarios across five broad simulation experiments (Table 1).

**TABLE 1:** The parameters simulated to evaluate the IAM within five simulation experiments for 30 scenarios

Parameter	Simulation 1: Variance in expert counts	Simulation 2: Expert data coverage	Simulation 3: Variation in misidentification	Simulation 4: Variation in abundance	Simulation 5: Variation in site occupancy
Scaling parameter for variance in expert counts (*k*)	**0, 0.25, 0.75, 1,** **1.5, 2**	1	1	1	1
Proportion of sites that expert data is available for	1	**0.1,0.2,** **0.4,0.6,** **0.8, 1**	1	1	1
Misidentification (*m*)	10	10	**0, 5,** **10, 15,** **20, 25**	10	10
Total abundance (∑*N_i_*)	2000	2000	2000	**500, 1,000, 1,500, 2000, 2500, 3000**	2000
Site occupancy (Ω)	0.8	0.8	0.8	0.8	**0.2, 0.4,** **0.6, 0.8,** **0.9, 1**

Note

Parameters that are tested within each experiment are in bold.

We first explored the effect of the quality of expert count data by simulating variance in observations. Given *σ^2^* = *N* × *k,* we varied *k* to explore scenarios where expert counts are perfect (*k* = 0), identical to Poisson distribution (*k* = 1) and then variance increases up to double that assumed by a Poisson observation error (*k* = 2). We also tested how the IAM performs with variation in the prevalence of expert data included, in abundance, and in levels of site occupancy.

Finally, we tested the performance of IAMs in response to manipulation of the misidentification parameter *m*, ranging from no misidentification to 25 false‐positive identifications per site.

For computational reasons, and because smaller sample sizes are more likely to be prone to identifiability problems (Kéry, 2018), all simulations assumed a conservative sample size of 20 sites and 10 replicates of citizen scientist counts. Additionally, the detection probability (*p*) was held at 0.8 for all scenarios. For each of the above scenarios, all other parameters remained constant to test the parameter of interest (Table 1).

The true site‐specific population sizes were simulated by first specifying probability of site occupancy. Occupied sites are then randomly selected from a binomial draw, and then for a specified total population size, a multinomially distributed random number vector is computed as true site abundance for occupied sites.

Citizen science data are subsequently simulated according to the relevant detection error and misidentification of that simulation and additional variation via a Poisson distribution across all sites and count replicates. Expert data are also subject to observation error via a Poisson distribution (although we test this assumption in Simulation Experiment 1), and when expert data are only available for a subset of sites it is removed randomly using a random number generator (see Appendix S1 for example).

### 2.8 Computational details

Models were specified within R version 3.6.1 (R Core Team, 2017), using the package R2WinBUGS version 2.1–21 (Sturtz et al., 2005) to call WinBUGS 1.4 (Lunn et al., 2000), within which the models were run, and from which results exported back to R (see Appendix S1). We used broad priors for each parameter as follows: uniform distributions U(0, 1) for detection probability and occupancy; uniform distributions (0, 40) for misidentification parameter; gamma distribution Gamma (1, 0.005) for site‐specific abundance. Preliminary simulations were assessed for convergence of the chains by visually checking mixing of the chains and more formally using the Brooks–Gelman–Rubin criterion (Brooks & Gelman, 1998). For each of the 30 different scenarios, we ran the model for 100 independent, simulated data sets. Following the initial trials for each simulation, we ran three chains of 20,000 with a burn‐in of 10,000 for each analysis and retained every 5th value, yielding a sample size of 6,000 iterations, from which full posteriors alongside summary values were stored. We note that thinning of chains is not always necessary but was required here to ease storage and memory demand across the 3,000 simulations.

### 2.9 Model assessment

For each scenario, we explore performance in terms of accuracy (proportion of simulations that capture the true value in their credible intervals), precision (widths of credible intervals), and bias (tendency for posterior distributions to lie above or below true values). The model provides per‐site abundance estimates (Appendix S1); however, for ease of testing, we use total abundance across areas for model testing ($\sum N_{i}$) that we refer to as *N*, alongside the detection and misidentification parameters.

## 3 RESULTS: SIMULATIONS

### 3.1 Simulation 1: Variance in expert counts

The IAM performed effectively under simulated scenarios in which the expert counts had variation less than or equal to their mean. When variation was equivalent to a Poisson distribution, accuracy was high resulting in accurate estimates for 95% of simulations. As variance in expert counts decreased, accuracy increased to 100%. However, increased variance in expert counts, over their mean (*k* > 1), resulted in reduced accuracy and precision in estimates of abundance and detection (Appendix S2 Figure S2), such that 74% of simulations were accurate when variance increased (*k* = 1.5) and only 64% of simulations were accurate when variance was double that expected from a Poisson distribution (*k* = 2). However, misidentification estimates were unchanged by variance in expert counts (Appendix S2 Figure S2) as misidentification can be estimated from the inclusion of expert data in unoccupied sites. This provides high accuracy of misidentification estimates regardless of the variation in expert counts.

### 3.2 Simulation 2: Coverage of expert data

**FIGURE 1:**
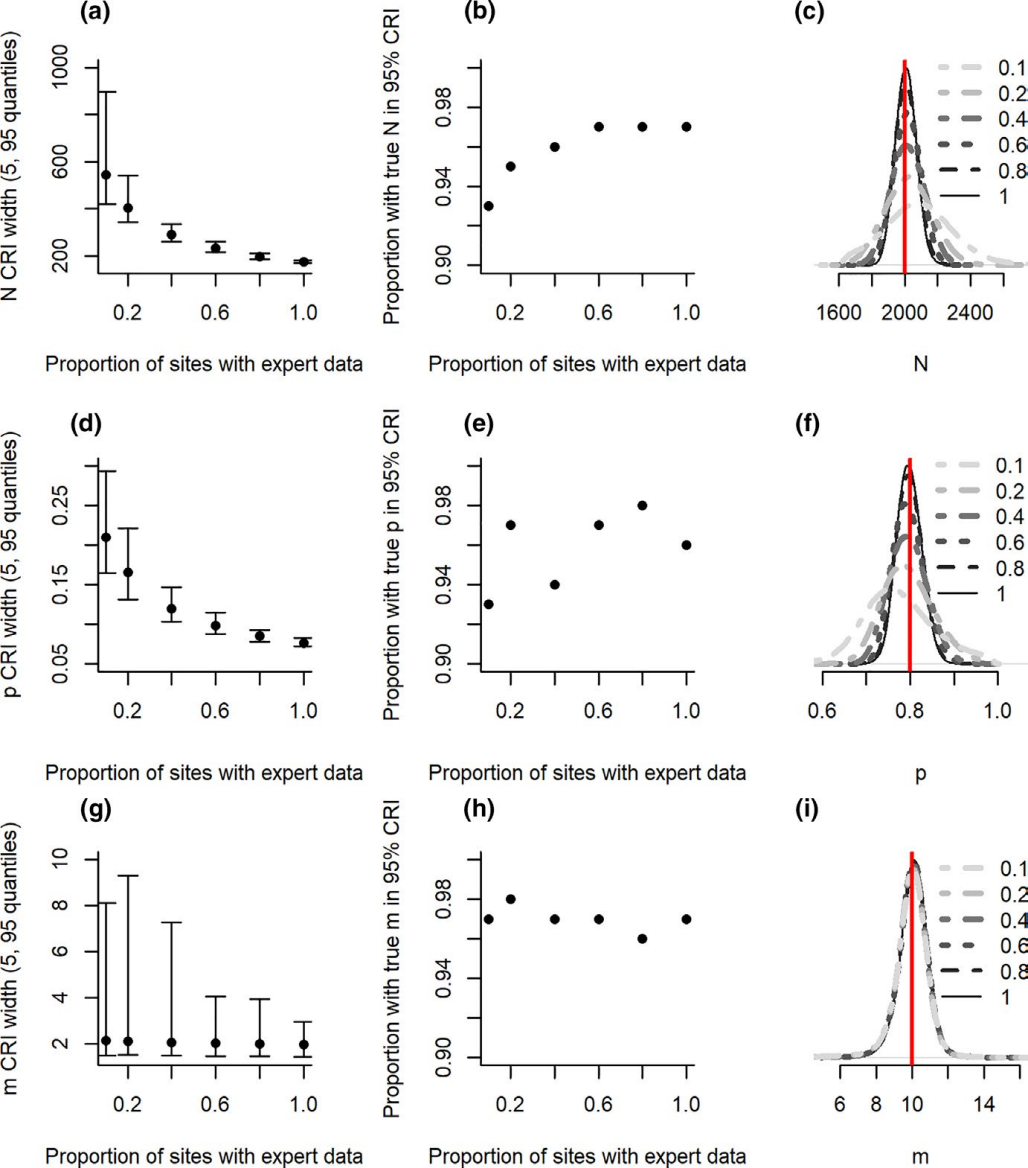
Precision (a, d, g), accuracy (b, e, h) and bias (c, f, I) of abundance (*N*; a–c), detection probability (*p*; d–f) and misidentification (*m*; g–i) from models in scenarios with different prevalence of expert data. Precision is measured as the width of the credible intervals (CRI). Points and whiskers show the 50%, 5%, and 95% quantiles, across replicate simulations, of the 95% CRI width for parameter estimates. Accuracy is measured here by the proportion of simulations where the true value is captured by the 95% CRI. Bias is observed as the full posteriors from all simulations

Accuracy remained high (>0.9) for all scenarios and parameters, likely due to reductions in precision in situations where there is low expert coverage (Figure 1). Although the model performed well across all scenarios, the slight skew of posterior distributions in abundance and detection parameters at low coverage indicate there was bias in some simulation runs, likely reflective of scenarios in which expert counts took place in sites where the target species was absent (Figure 1). The inference of misidentification was not biased by the amount of expert coverage, despite reductions in precision with limited expert data (Figure 1).

### 3.3 Simulation 3: Misidentification bias in citizen scientist counts

**FIGURE 2:**
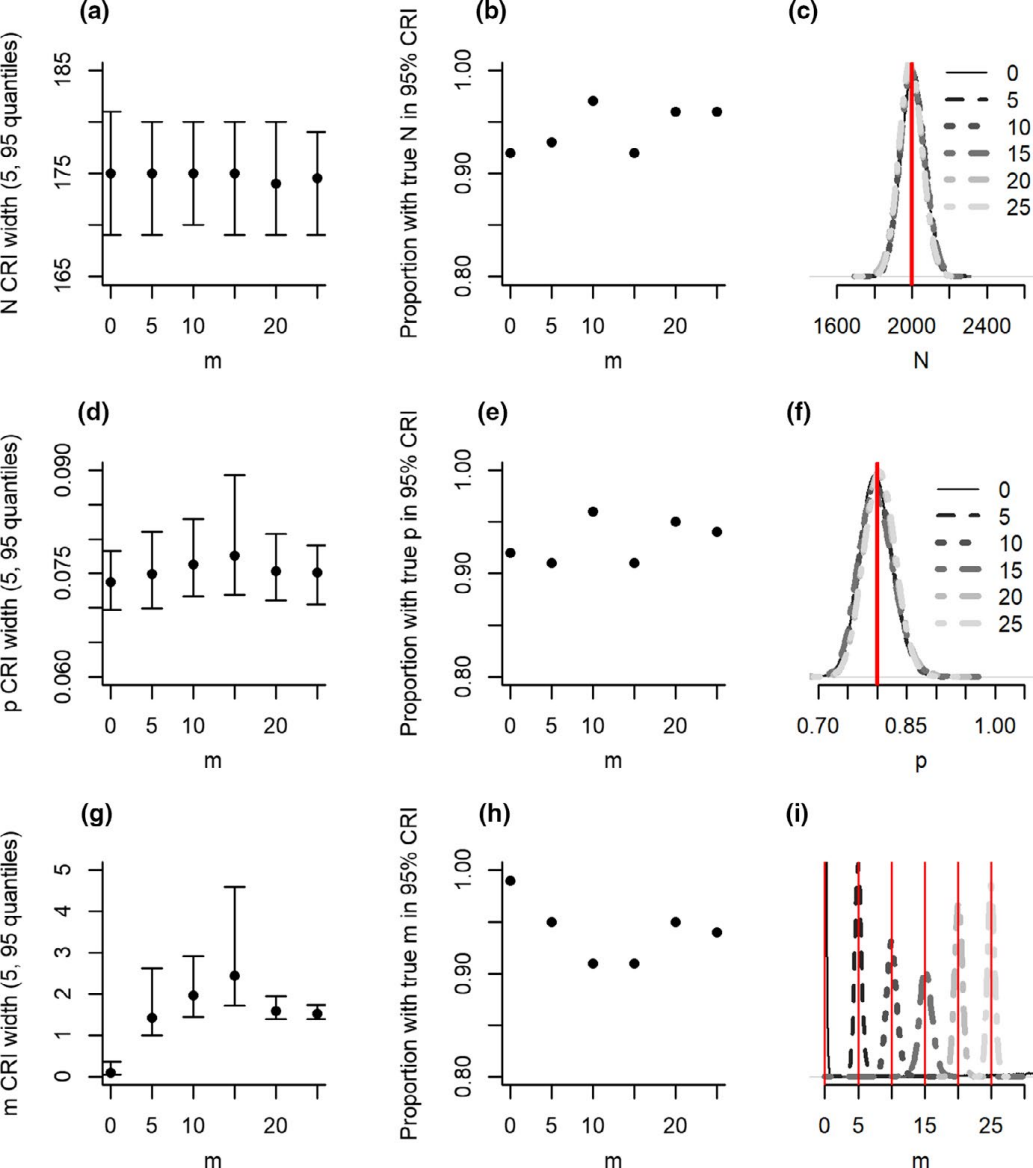
Precision (a, d, g), accuracy (b, e, h) and bias (c, f, I) of abundance (*N*; a–c), detection probability (*p*; d–f) and misidentification (*m*; g–i) from models in scenarios with different levels of per‐site misidentification. Note, when *m* = 0 accuracy was defined as true when the upper CRI limit < 0.5. Additional figure details can be found in the Figure 1 legend

The IAM performed without bias under simulated scenarios with different levels of misidentification (Figure 2). All parameters had high accuracy (>90%) regardless of the magnitude of misidentification. Precision of misidentification estimates increased with low and high levels of misidentification, but remained constant for detection probability and abundance.

#### 3.3.1 Simulation 4: Abundance

**FIGURE 3:**
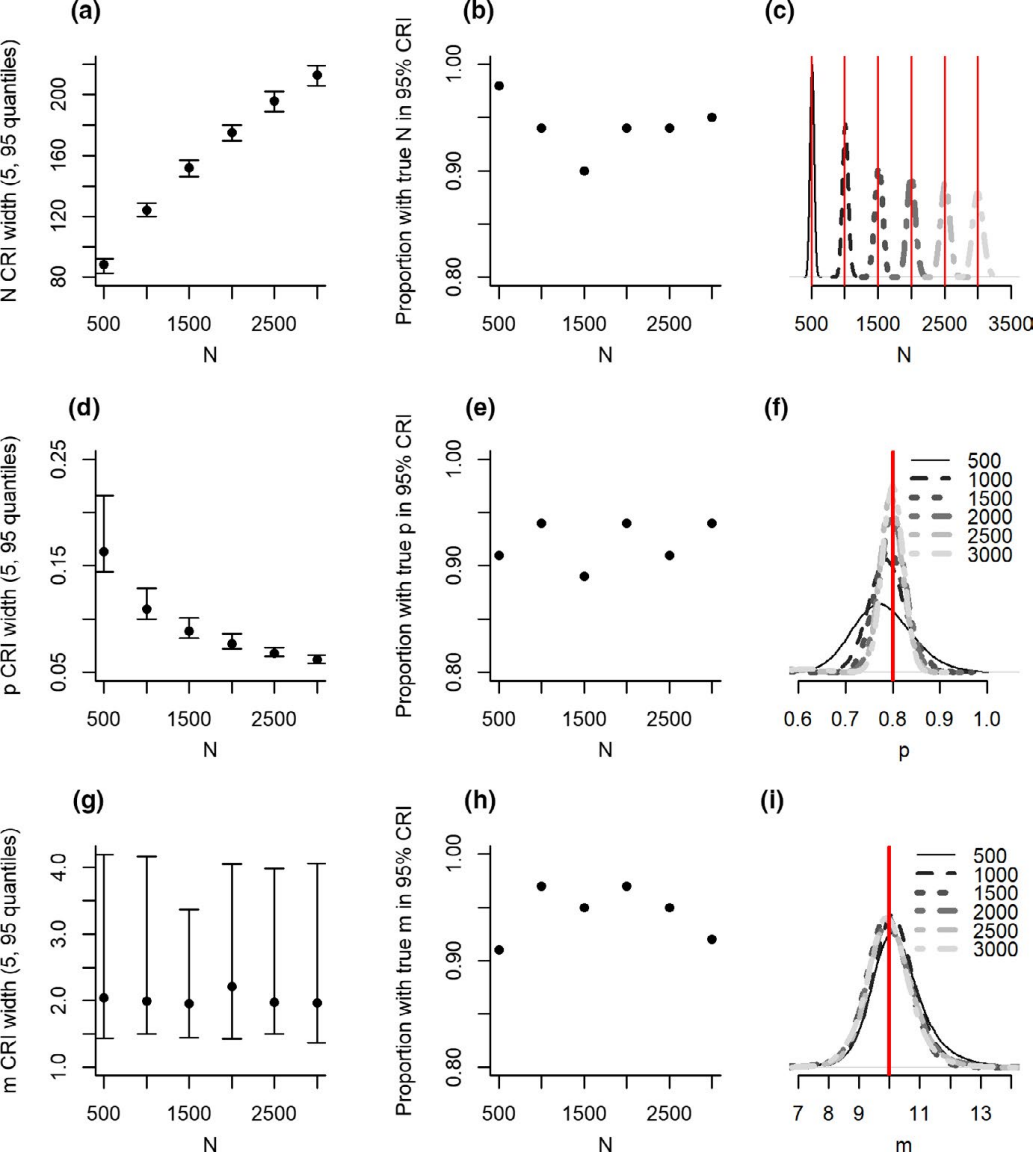
Precision (a, d, g), accuracy (b, e, h) and bias (c, f, I) of abundance (*N*; a–c), detection probability (*p*; d–f) and misidentification (*m*; g–i) from models in scenarios with different abundance. Additional figure details can be found in the Figure 1 legend

Abundance estimates had high accuracy and no bias regardless of the size of the underlying sample population. Precision in estimates reduced with population size, as would be expected with variability increasing with abundance (Figure 3). In contrast, detection probability had the lowest precision at lower population sizes (Figure 3). We find here a slight bias in detection at low population abundance. This bias was not observed in the misidentification parameter.

#### 3.3.2 Simulation 5: Occupancy

**FIGURE 4:**
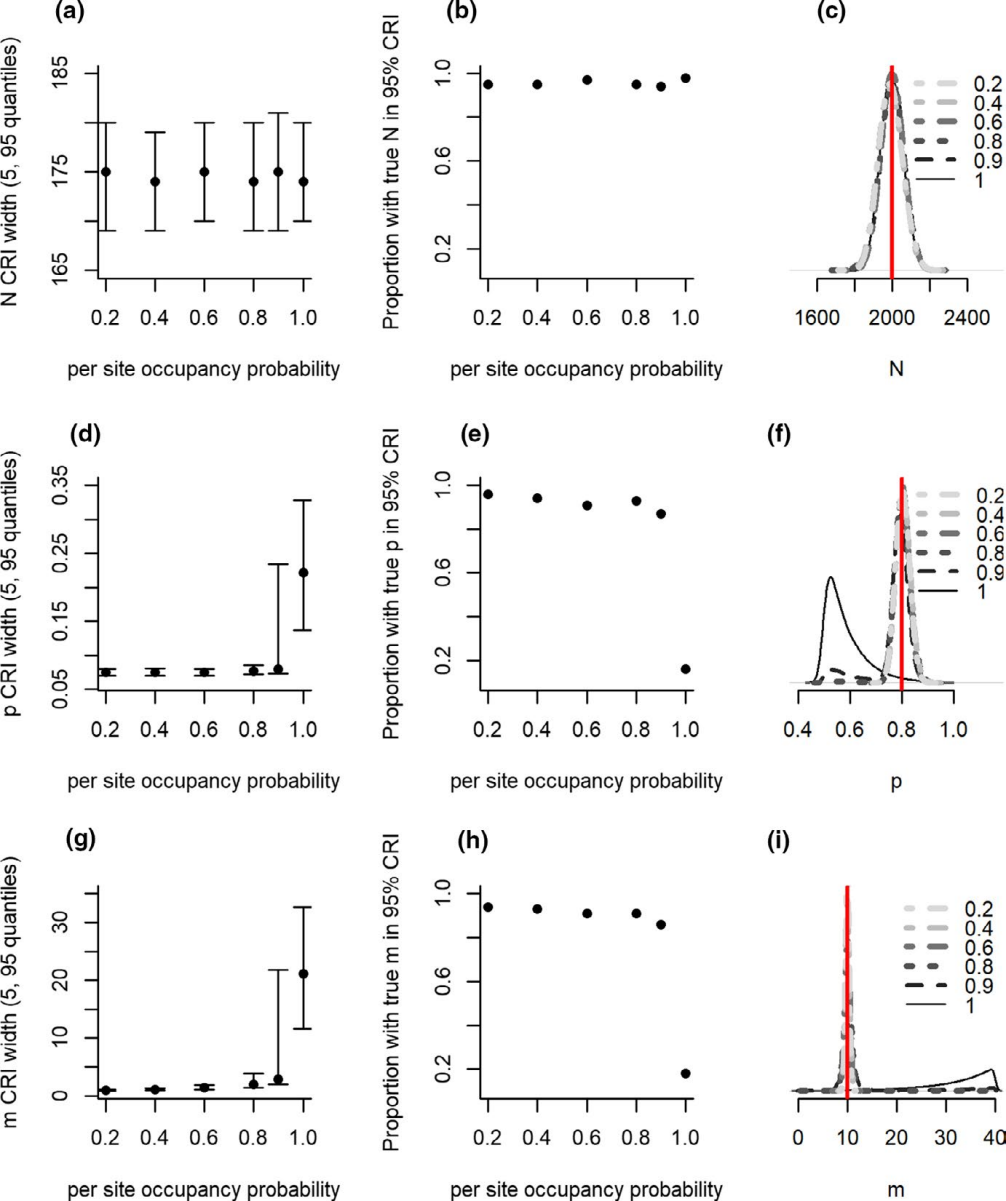
Precision (a, d, g), accuracy (b, e, h) and bias (c, f, I) of abundance (*N*; a–c), detection probability (*p*; d–f) and misidentification (*m*; g–i) from models in scenarios with different probabilities of site occupancy. Additional figure details can be found in the Figure 1 legend

The IAM had high accuracy, low bias, and a constant degree of precision in estimates of abundance, regardless of the occupancy of the population (Figure 4a,b). However, misidentification and detection probability were only identifiable when occupancy was less than 100% (Figure 4d–f). The IAM relies on some unoccupied sites to infer misidentification.

### 3.4 Case study: Unowned cats

To illustrate the biases risked by ignoring misidentification and to show that IAMs remove these biases, we analyze data from a study of unowned cats in an urban area. Data were collected as part of a wider community outreach program in Bulwell, Nottingham, UK called “Bulwell Cat Watch”. The project itself combines community engagement with neutering and rehoming operations with the aim to bring about human behavior change and control cat numbers (full details published elsewhere see (McDonald & Clements, 2019; McDonald et al., 2018). Bulwell was chosen as an area where unowned cats were thought to be prevalent, based on previous charity work in the community. Data used here are taken from the first 12 months of operations (September 2016–August 2017).

#### 3.4.1 Study species

Domestic cats are an intrinsic component of human society in the UK, with over 10 million owned cats (Murray et al., 2015). Many owned cats have accidental litters (Welsh et al., 2014) and a large number of cats become abandoned, relinquished, or lost each year (Clark et al., 2012; Stavisky, 2014; Stavisky et al., 2012); thus, unowned cats are also ubiquitous across many urban ecosystems. Effective and humane management of unowned cats (that are comprised of stray and feral subgroups) may function to improve their welfare and control numbers; however, there is limited understanding of their abundance in urban areas.

Although identifying unowned domestic cats is valuable for management purposes, their identical physiologies to owned cats means accurate identification is a challenge for local residents (McDonald & Clements, 2019). Traditional wildlife monitoring approaches are also unable to differentiate between these key cat subgroups (Elizondo & Loss, 2016; Flockhart et al., 2016; Hand, 2019), and researchers rarely have access to private spaces in built‐up areas (Hand, 2019; Kilgour et al., 2017). Consequently, a community engagement approach is needed to improve accessibility. Additionally, the benefits associated with an animal welfare approach are necessary for both positive public engagement, to provide appropriate management for these cats, and accurate recognition of unowned cats, with identification protocols commonplace within welfare organizations.

#### 3.4.2 Citizen Science data

Two different forms of CS data were collected.

1. Survey data: The first consisted of an initial cross‐sectional random‐sample door‐to‐door survey carried out with approximately 10% of households (*n* = 776). At that stage, residents were asked how many cats they know of locally and how many they think were owned in the form of a multiple‐choice question with the following options: none, 1–2, 3–4, 5–9, 10, or more, from which the number of unowned cats was derived. When a range was selected, the central value was taken; for ten or more, we used 15 (the average from reports when 10 or more was specified). Location data were available for 695 survey responses, within which there were estimates of 1,318 unowned cats.
2. Report data: Throughout the project, residents were able to report unowned cats in their area directly via social media or through a Cat Watch mobile application. During the study period, 241 reports were received reporting on the locations of 965 unowned cats.

#### 3.4.3 Expert data

In addition to the resident reports of unowned cats, the community team (CT) recorded when and where an unowned cat was found or where unowned cats were not present. These data are considered of higher quality, due to the ability of the CT to correctly identify an unowned cat and with no risk of double counting the same individual. Unowned cats can be either stray or feral. Protocols to accurately identify a stray cat included: scanning for a microchip, attaching a paper collar to notify potential owners, advertising online, door‐to‐door notifications, local posters and contacting other animal welfare organizations, including veterinary practices. If no owner was found during this process, it was identified as unowned.

Feral cats were more likely to be identified via behavioral means, as they have not been socialized to humans, they will be more fearful and will not approach humans (Gosling et al., 2013). If they have already been neutered, they may also have their left ear “tipped”.

During the study period, there were 145 records from the CT, reporting on the location of 117 confirmed unowned cats.

All three of these data sources provided detailed location data (postcodes and/or addresses) enabling geo‐referencing of unowned cat location data.

#### 3.4.4 IAM

Estimates of unowned cats obtained from the public are prone to biases due to misidentification with the owned cat population and duplicate sightings from closely situated residents.

To account for duplicate sightings, the CS data required clustering to account for neighbors in close‐proximity reporting the same cats and for a certain degree of cat movement. There is limited understanding of urban unowned cats in the UK; however, studies of urban unowned cats in other areas indicate home range sizes between 3.7 and 10.4 ha for urban areas (Pillay et al., 2018; Tennent & Downs, 2008). Studies on unowned cats in the UK indicate that home ranges vary between 10 and 15 hectares (Page et al., 1993). We assume a maximum 20 ha home range, equivalent to a circular area with a diameter of 504 m. Consequently, we apply a 500 m cluster function in R (R Core Team, 2017) that derives clusters of cat sightings that are within 500 m of each other. The data set of the CS data (survey and reports) consisted of replicate counts within each cluster. The effect of violating this assumption (i.e., reporting them as duplicate sightings when they are not) would result in bias in the observation parameters, not estimates of the cats themselves, which are also inferred from the expert data.

We ran two separate IAMs: (a) integrating survey data with expert data and (b) integrating report data with expert data. Expert data were not available for all sites, 75% expert coverage for the survey data (21 out of 28 sites) and 91% expert coverage for the report data (20 out of 22 sites).

#### 3.4.5 Model assessment

Assessing model fit of Bayesian hierarchical models applied to field data is complex, with each proposed solution associated with its own strengths and weaknesses (Conn et al., 2018). We discuss our approach here. However, with a wide range of approaches (Hooten & Hobbs, 2015), this will be an important area of consideration as the model is developed further, especially if users wish to compete rival models to help test the importance of parameters and hypotheses.

Our model‐checking procedures differ between our simulation study and our empirical case study. The simulations enjoy knowledge of the true values of the parameters, allowing us to assess the performance of the model, this is a useful tool to check model performance in scenarios that represent the field system of interest. For our case study, first, we examined key indicators of fit including identifiability of parameters, despite vague priors, and convergence of MCMC chains. Convergence of multiple chains is required to check for multiple posterior modes. Second, we test the influence of modeling false positives by comparing IAM results to a traditional N‐mixture model that does not include expert counts and only considers detection probability. Comparison of the abundance estimate between the two approaches indicates whether false positives are having a strong influence on the system (i.e., a lack of overlap in 95% CRI of *N* between the two approaches). Third, our case study includes two forms of CS data. If biases are accurately accounted for and true abundance is identifiable, we would expect there to be overlap in the abundance estimate when these two models are run separately (i.e., an overlap in 95% CRI of *N* between the two IAMs). Fourth, we ran further cross‐validation on our IAM by removing one expert data point at a time, conducting the analysis, and checking how model predictions match up. The rationale being if there were heterogeneity unaccounted for and/or specific sites that may be outliers worthy of investigation, then the removal of those data points would result in outcomes inconsistent with the overall model (i.e., a lack of overlap in 95% CRI of model parameters *N*, *m*, and *p*). Finally, we simulated data sets structured according to the raw data and parameterized using the model estimates to check model performance under similar scenarios (Appendix S3).

#### 3.4.6 Results of case study

Our results indicate that relying on CS data alone, either through simple summation or via traditional N‐mixture modeling approaches, can vastly inflate estimates of the number of unowned cats. Here, we found between a sixfold and 23‐fold increase in cat estimates depending on the method applied (Table 2).

**TABLE 2:** Total number of cats calculated via different methods and associated observation parameters when estimated through modeling approaches

Method of calculation	Parameter		
	Total number of cats	Detection probability	Misidentification
Sum across all survey data	1,318	NA	NA
Sum across all reports	965	NA	NA
Sum of all expert data	117	NA	NA
Apply N‐mixture model to survey data	1,261 (1019, 1605)	0.04 (0.03,0.05)	NA
Apply N‐mixture model to report data	3,414 (1576, 6468)	0.03 (0.01, 0.05)	NA
**Apply IAM to survey data and expert data**	**151 (126, 180)**	**0.21 (0.17, 0.27)**	**0.75 (0.64,0.87)**
**Apply IAM to report data and expert data**	**143 (114,190)**	**0.14 (0.10, 0.19)**	**2.54 (2.19, 2.91)**

Note

The mean and 95% CRI are stated for model‐derived estimates. The model results obtained from the IAMs and presented in the main text are shown in bold.

**FIGURE 5:**
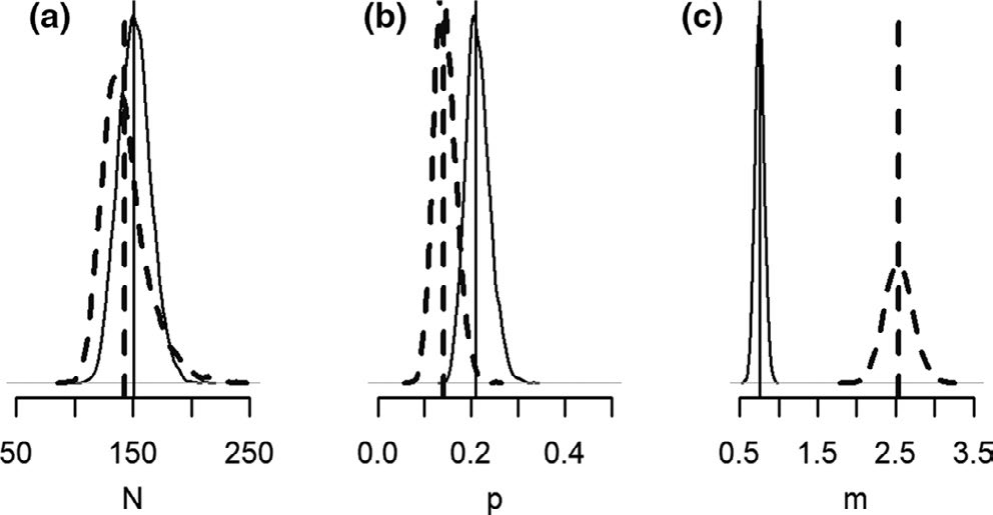
The posterior distributions of total unowned cat abundance (*N*), detection probability (*p*), and misidentification (*m*) from an IAM integrating expert data with CS data obtained from a survey approach (solid line) and reporting approach (dashed line), along with their mean. Note, that although the detection probability and misidentification varied between survey approaches, the total abundance was similar

IAMs accounted for the differing biases within the CS data. Within our case study, data collected via reports were more prone to misidentification of owned cats and underdetection of unowned cats, compared to information collected via a random sample survey (Table 2 and Figure 5). Having removed these biases, IAMs provided similar total abundance estimates regardless of the CS data included, with overlap in posteriors (Figure 5), estimating a mean of 143 and 151 unowned cats depending on the underlying CS data (Table 2). The average number of cats per site was six (range zero to 26) for reports and five (range zero to 23) for survey data. Model results were robust against further model validation that applied a leave‐one‐out approach to expert data (Appendix S3; Figures S3 and S4). Additionally, the IAM performed well on simulations parameterized to represent this field system (Appendix S3: Figures S5 and S6).

## 4 DISCUSSION

Citizen science provides a valuable tool for collecting large quantities of data across spatial and temporal scales not otherwise achievable, but current modeling approaches come with assumptions that may often be violated, particularly that false‐positive counts should not occur. Our simulation study and case study illustrate that abundance estimates are prone to be highly inflated if inference is based on traditional modeling approaches when false positives are present. Such misleading estimates would likely hinder or harm conservation and management programs. We have introduced a new class of models for the Bayesian inference of abundance based on the integration of potentially lower quality CS data (prone to misidentification) with high‐quality expert counts from a subset of locations. IAMs perform well in terms of precision, accuracy, and lack of bias across a wide range of simulation experiments. Our case study illustrated the applicability of IAMs to real‐world data, offering a solution to the problem of misidentification bias. We discuss the benefits and limitations of this framework, alongside possible avenues of development of this new toolkit.

IAMs provide an ideal opportunity to integrate data sets accounting for different biases between citizen scientists and experienced surveyors to help provide unbiased estimates of the abundance of important animal populations. This model does not require error‐free expertise, increasing the applicability of this approach. Data derived from expertise in our model framework are still subject to an observation error similar to that commonly used in state‐space models of population counts where it is applied to estimate temporal measures of abundance (Belant et al., 2016; Iijima et al., 2013; Westcott et al., 2018).

However, we do find that increased variability in expert counts above that expected from a Poisson distribution can result in reduced precision and accuracy; therefore, the applicability of these models necessitates assumptions of limits on the variability in expert counts. A key benefit of IAMs is they can perform accurately with just 10% expert coverage, indicating resources would be best placed ensuring high‐quality data in a subset of sites, rather than increasing site coverage at the expense of reduced quality.

IAMs also infer probabilities of detection and misidentification, which might themselves be of interest to survey managers and to citizen scientists themselves. Indeed, testing of multiple methods of data collection is commonplace (Belant et al., 2016; Molinari‐Jobin et al., 2012; Sawaya et al., 2012; Stober & Smith, 2010), and as our case study illustrated, IAMs provide a framework to model and assess biases in these different methods. An understanding of detection error across approaches can help prioritize future monitoring strategies and improve efficiencies. Additionally, participation in CS programs can increase identification skills (Jordan et al., 2011). Therefore, inference of misidentification might help citizen scientists graduate from amateur to expert status, with ratings systems already used in some programs (Clow & Makriyannis, 2011).

A limitation of the IAM is its reliance on inference from unoccupied sites to accurately estimate detection bias. At 100% site occupancy, detection and misidentification parameters are confounded and consequently unidentifiable, while abundance estimates remain accurate. This limits the wider application of IAMs to estimate observation bias in situations where target and nontarget individuals consistently reside together. However, improved model inference may be included in other ways such as informative priors, which can resolve identifiability issues in other areas (McDonald & Hodgson, 2018) or integration of multiple survey approaches that often have different detectability (Sawaya et al., 2012), potentially increasing our power to detect biases. This area warrants further development.

A method for robust inference of abundance is vital to aid decision‐makers, in situations where binary occupancy information is not sufficient (Johnston et al., 2015). Unbiased estimates of abundance will help conservation managers to make evidence‐based decisions for the prioritization of management interventions. Changes in abundance contribute to indices of endangerment in the IUCN Red List; however, financial constraints can restrict the use of abundance surveys (Joseph et al., 2006). We believe that the use of CS could help to move many species from the “Data Deficient” category to a credible category of conservation concern. An additional benefit of CS is not only its reach but also the ability to collect data from areas not otherwise accessible such as gardens (Lye et al., 2012) or behind homes and businesses as our case study illustrated. Therefore, increasing our ability to make robust inferences from potentially previously unexplored communities and habitats.

As with all models, IAMs may not be appropriate for all data sets. The applicability and development of IAMs are best explored via simulations that are tailored to the specific field systems and data collection approaches. Simulations, including those tailored to our case study, highlighted that abundance estimates were consistently accurate across a wide range of scenarios. However, we note that at low expert coverage there was bias in some simulation runs, likely reflective of inadequate coverage across occupied and unoccupied sites. Consequently, where low occupancy is thought to occur researchers are encouraged to consider both their methodological approach by increasing the quantity of expert data, but also assess the degree of expert data required to identify parameters relevant to their specific scenario through model simulations. Additionally, where there is a strong knowledge base, sensible bounded priors on detection parameters would likely improve precision further. Indeed, early studies have recommended incorporating expert consensus within prior information as a cost‐effective way of improving confidence in abundance predictions (Martin et al., 2005) and may be worth considering to improve inference in low occupancy scenarios. Another assumption made by our IAMs is that expert counts are made in an unbiased set of sites, such that sites surveyed by experts do not differ in some systematic way from those surveyed by citizen scientists. Not correcting for such bias when it is present has been shown to reduce the benefits of integrating data within distribution models (Simmonds et al., 2020). Testing what happens if these assumptions are violated within IAMs would deepen our understanding of the performance of these models.

We have presented methods here for constant, time‐invariant IAMs, but one value of our hierarchical modeling approach is that it permits direct extension to more sophisticated models. The inclusion of site‐specific covariates and temporal extensions are all possibilities. Indeed, this development would be welcome, reducing assumptions of homogeneity across sites, which may result in bias estimates, as has been found for incorrectly fitted N‐mixture models (Knape et al., 2018). Additionally, there is potential scope to explore direct ways of weighting expert data as we understand more about the different impacts of weighting schemes in the constantly growing area of data integration (Fletcher et al., 2019). Finally, one of our key assumptions is that misidentification is independent of abundance of the target species. This assumption ignores a suite of ecological patterns, for example co‐occurrence of similar species in suitable habitats, or competitive exclusion of similar species. These ideas suggest a large number of possible investigations, but we hope that the adaptability of this model framework sees greater uptake by ecologists and animal welfare scientists to develop bespoke models and perform integrated analysis tailored to questions and biases in their field systems of interest.

## 5 CONCLUSION

With citizen science data so abundant, it is important to understand and address the potential biases concerning their use. Integrated Abundance Models address the challenges posed by CS data, allowing ecologists and animal welfare managers to better harness this immense resource when monitoring animal populations. Integrating data sources of differing quality improves precision of abundance estimates by accounting for misidentification biases. We encourage future studies to use IAMs when false positives are thought to occur. The framework described is adaptable and we hope it provides a useful introduction to the concept to allow further optimization of the approach, tailoring to specific systems, and greater use of CS data.

## CONFLICT OF INTEREST

None declared.

## AUTHOR CONTRIBUTIONS

**Jenni L. McDonald:** Conceptualization (lead); data curation (lead); formal analysis (lead); investigation (lead); methodology (lead); project administration (lead); resources (lead); software (lead); writing–original draft (lead); writing–review and editing (equal). **Dave Hodgson:** Methodology (supporting); writing–original draft (supporting); writing–review and editing (equal).

## ETHICAL APPROVAL

For the simulation study, data were completely simulated which did not require approval from an ethics committee. The case study example used was based on a subset of data collected as part of a study that had been approved by University of Bristol Faculty of Health Science Research Ethics Committee approval number 38661.

## Supporting information

Appendix S1

Appendix S2

Appendix S3

## Data Availability

All simulation code supporting the conclusions of this article, including the R script for simulating abundance data and analyzing the data in R2Winbugs, is available in Appendix S1. The data that support the findings of the case study are part of an ongoing project collated and managed by Cats Protection. Data can be available from the corresponding author upon reasonable request and with permission of Cats Protection, with necessary redactions of identifying information such as precise geographic coordinates.
