# Supplementary material for: Counting Cats: The integration of expert and citizen science data for unbiased inference of population abundance
Source: Ecol Evol. 2021 Apr 2;11(9):4325–38. doi: 10.1002/ece3.7330 (PMC8093703; doi:10.1002/ece3.7330)
Supplement: Supplementary file 1 — Appendix S1 [file ECE3-11-4325-s003.docx]

**Appendix S1**

**Integrated Abundance Models:**

**Demonstration of Simulations and Model Code**

1. *Illustration of simulated data inputs and model outputs of a IAM*

An example of a simulated dataset (*p*=0.8, *m_i_*=5, ∑*N_i_*= 2000, Ω=0.8) with expert counts available for 50% of the sites, alongside model outputs is detailed in Table S1. The model provides per site abundance estimates and total abundance across areas can be used for model testing. In the example below total abundance was estimated to be 1971 (95% CRI 1841, 2107), detection 0.81 (95% CRI 0.76, 0.87) and per site misidentification rate 9.8 (95% CRI 8.8, 10.7) similar to the simulated truth.

Table S1. Example simulated data set, that includes citizen science data subject to misidentification bias for each site *i* 1,2….20 and replicate counts *j* 1,2,….10 (*y_i,j_*), expert data for 50% of these sites (*w_i_*) alongside site specific model estimates

| site | True  N*_i_* | Simulated data for model input | | | | | | | | | | | Model output | |
| --- | --- | --- | --- | --- | --- | --- | --- | --- | --- | --- | --- | --- | --- | --- |
|  |  | *y_i,1_* | *y_i,2_* | *y_i,3_* | *y_i,4_* | *y_i,5_* | *y_i,6_* | *y_i,7_* | *y_i,8_* | *y_i,9_* | *y_i,10_* | *w_i_* | mean | CRI |
| 1 | 0 | 9 | 6 | 6 | 11 | 9 | 13 | 5 | 5 | 11 | 5 | NA | 0 | (0, 0) |
| 2 | 137 | 106 | 133 | 141 | 109 | 143 | 125 | 111 | 114 | 135 | 117 | 132 | 138.7 | (127.6, 150.6) |
| 3 | 123 | 104 | 108 | 117 | 103 | 107 | 83 | 127 | 99 | 128 | 90 | NA | 119 | (108, 131) |
| 4 | 0 | 8 | 12 | 12 | 9 | 10 | 17 | 11 | 9 | 16 | 11 | 0 | 0 | (0, 0) |
| 5 | 129 | 126 | 118 | 105 | 98 | 113 | 108 | 120 | 117 | 136 | 133 | NA | 132.4 | (120.7, 145) |
| 6 | 124 | 113 | 110 | 118 | 124 | 116 | 87 | 118 | 105 | 108 | 106 | 108 | 122 | (111.5, 132.5) |
| 7 | 128 | 121 | 111 | 100 | 118 | 95 | 105 | 116 | 93 | 116 | 123 | NA | 123.1 | (111.9, 135) |
| 8 | 0 | 8 | 12 | 8 | 12 | 3 | 8 | 11 | 11 | 11 | 8 | 0 | 0 | (0, 0) |
| 9 | 121 | 92 | 110 | 102 | 108 | 85 | 92 | 111 | 113 | 101 | 102 | 121 | 113.9 | (104.3, 124.4) |
| 10 | 113 | 103 | 83 | 99 | 123 | 92 | 103 | 86 | 99 | 111 | 107 | NA | 111.8 | (101.1, 123.1) |
| 11 | 128 | 107 | 95 | 122 | 123 | 114 | 109 | 130 | 117 | 133 | 113 | 128 | 130.7 | (120, 142.1) |
| 12 | 141 | 126 | 120 | 122 | 98 | 132 | 108 | 103 | 119 | 121 | 126 | 140 | 133.3 | (122.5, 145.2) |
| 13 | 128 | 126 | 117 | 102 | 101 | 110 | 117 | 128 | 122 | 108 | 122 | NA | 129.7 | (118, 142) |
| 14 | 0 | 12 | 13 | 12 | 9 | 11 | 9 | 8 | 9 | 9 | 9 | 0 | 0 | (0, 0) |
| 15 | 119 | 99 | 98 | 100 | 99 | 83 | 101 | 84 | 99 | 101 | 93 | NA | 105.8 | (95.9, 116.4) |
| 16 | 130 | 140 | 108 | 101 | 122 | 107 | 129 | 113 | 112 | 104 | 99 | NA | 127.6 | (116.4, 140.1) |
| 17 | 118 | 96 | 97 | 109 | 116 | 115 | 113 | 96 | 113 | 124 | 91 | NA | 119.5 | (108.6, 131.3) |
| 18 | 108 | 107 | 97 | 97 | 114 | 85 | 85 | 97 | 86 | 112 | 95 | 97 | 106.6 | (97.4, 116.5) |
| 19 | 132 | 122 | 121 | 119 | 106 | 129 | 109 | 105 | 118 | 101 | 121 | 144 | 131.3 | (120.7, 142.5) |
| 20 | 121 | 105 | 131 | 118 | 105 | 118 | 114 | 108 | 96 | 116 | 107 | NA | 125.5 | (114, 137.9) |

1. *IAM R Code*

#Data simulation

nsites=20 #number of sites

Ry=10 #number of replicate counts in amateur data set

omega=0.8 #occupancy

totalN=2000 #total abundance across sites

p=0.8 #detection probability

m=10 #misidentification of non-target species per site

y=array(dim=c(nsites,Ry))

mu=array(dim=c(nsites,Ry))

z<-rbinom(nsites,1,omega)#occupancy

nos<-nsites-length(z[z==0])#number of occupied sites

n<-rep(0,nsites)

n[z>0]<-rmultinom(n=1,size=totalN,prob=rep(1/ nos, nos))#split total abundance to unique sites

#Simulate expert data subject to observation error (w)

w=rep(0,nsites)

w=rpois(nsites,n)# model observation error in expert counts

#w=rnorm(nsites,n,sqrt(n))##number, mean, sd- equivalent to poisson . This alternative normal #approximation can be used to model over and under dispersion in expert counts.

#Remove random sample of 50% of sites from expert data

remove<-sample(1:nsites,nsites*.5,replace=FALSE)

w50<-w

w50[remove]<-NA

#Remove from occupancy also

z50<-z

z50[remove]<-NA

#Simulate amateur data subject to detection, misidentification and Poisson error (y)

for(i in 1:nsites){

mu[i,]<-(n[i]*p)+m

y[i,]<-rpois(Ry,lambda=mu[i,])

}

# Misidentification IAM

sink("MisidentificationIAM.txt")

cat("

model {

# Priors

omega~dunif(0,1) #occupancy

p~dunif(0,1) #detection

m~dunif(0,40)#misidentification -4 x true value

#Likelihood

#observation model expert counts

for (i in 1:nsites){ #Loop over R sites

z[i]~dbern(omega)

lambda[i]~dgamma(1,0.005)

N[i]<-z[i]*lambda[i]

W[i]~dpois(N[i])

#Observation model for replicated amateur counts

for (j in 1:R){

y[i,j]~dpois(Y[i,j])

Y[i,j]<-(N[i]*p)+m

}

}

##derived quantities

totalN<-sum(N[]) }

",fill=TRUE)

sink()

win.data<-list(y=y, W=w50, R=ncol(y), nsites=nsites,z=z50)

#Can model without occupancy specified e.g.

#win.data<-list(y=y, W=w50, R=ncol(y), nsites=nsites)

params<-c( "p","totalN","N","m")

ni=20000

nt=5

nb=10000

nc=3

#Model runs with initials not specified however, including initials can prevent winbugs trap messages in some scenarios

out1<-bugs(win.data,inits=NULL,params,"MisidentificationIAM.txt",n.chains=nc,n.thin=nt,n.iter=ni,n.burnin=nb,debug=TRUE,bugs.directory = bugs.dir, working.directory = getwd())

modeloutcome<-out1$summary
