# Supplementary material for: Counting Cats: The integration of expert and citizen science data for unbiased inference of population abundance
Source: Ecol Evol. 2021 Apr 2;11(9):4325–38. doi: 10.1002/ece3.7330 (PMC8093703; doi:10.1002/ece3.7330)
Supplement: Supplementary file 2 — Appendix S2 [file ECE3-11-4325-s002.docx]

**Appendix S2**

**Integrated Abundance Models:**

**Extra Model Testing Results**

1. **N-mixture vs IAM**

We demonstrate the potential bias caused by assumptions of no false positives occurring.

*IAM compared to N-mixture model*

We demonstrated the potential bias of assumptions of no false positives due to misidentifications when they occur, by 100 simulation runs (*p*=0.6, *m*=5, ∑*N_i_*= 2000, Ω=0.8) comparing median posterior values and accuracy of N-mixture models with an IAM, where there is 50% and 100% expert coverage. When misidentification is present an N-mixture model is unable to account for the number of false positives resulting in slightly inflated abundance estimates, and increased, inaccurate detection probability. There was low accuracy across parameters in the N-mixture model (Fig. S1).


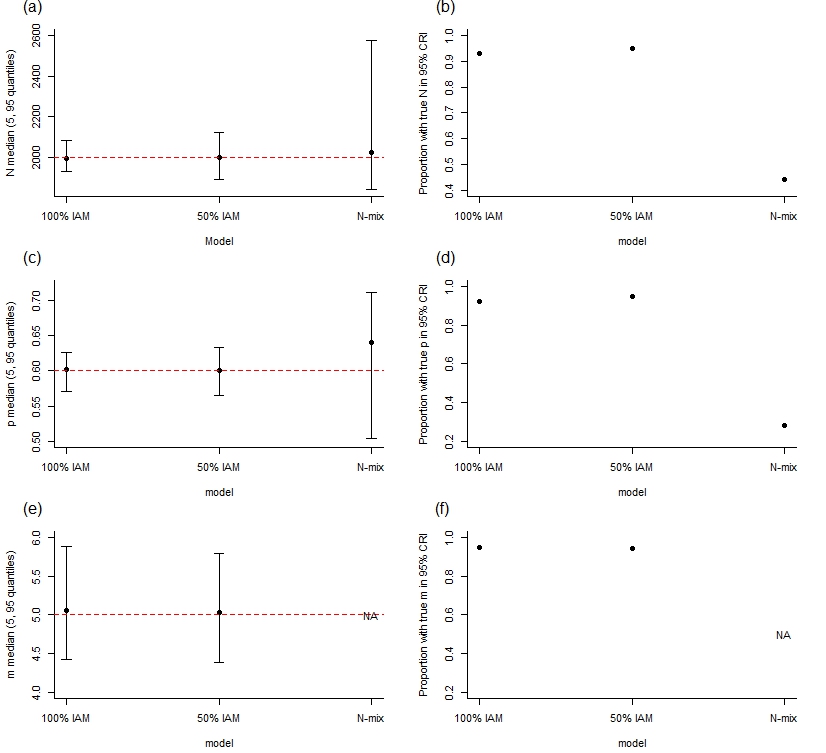


Figure S1. Comparison of Misidentification IAM model and N-mix models for analysing data with false positives due to misidentification (a,c, e) median of the posteriors. Points and whiskers show the 50%, 5% and 95% quantiles, across replicate simulations, (b,d,f) Accuracy is measured here by the proportion of simulations where the true value is captured by the 95% CRI.

1. **Poisson vs Normal Observation model: variation in expert counts**


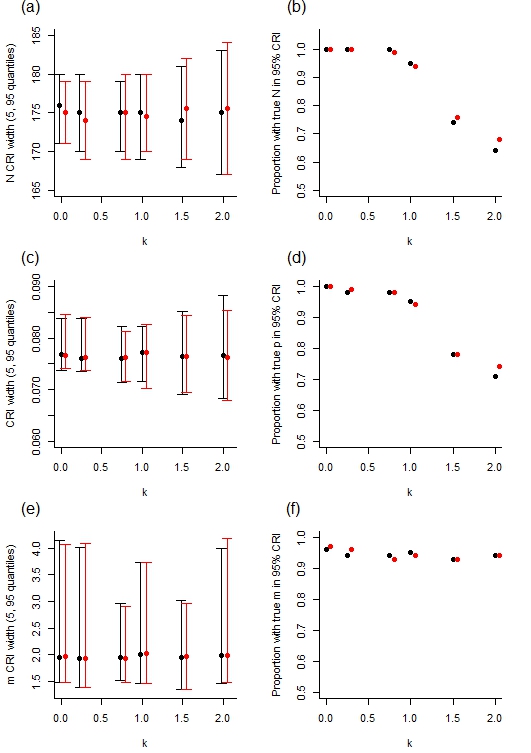


Figure S2. Comparison of a Poisson (black) and Normal (red) observation model under varying degrees of variation (k) in expert counts in an IAM from Simulation 1 see table 1 in main manuscript. The Normal model permitted variation over and above a Poisson observation model (priors *k*~dunif(0,3)), but did not change findings.
