# Supplementary material for: Counting Cats: The integration of expert and citizen science data for unbiased inference of population abundance
Source: Ecol Evol. 2021 Apr 2;11(9):4325–38. doi: 10.1002/ece3.7330 (PMC8093703; doi:10.1002/ece3.7330)
Supplement: Supplementary file 3 — Appendix S3 [file ECE3-11-4325-s001.docx]

**Appendix S3**

**Integrated Abundance Models:**

**Case study further testing**

1. **Case study model validation- a leave-one-out approach to expert data**


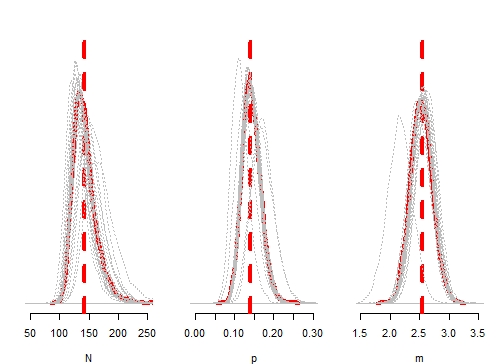


Figure S3. Validation analysis of IAM applied to expert data and report data, illustrating the impact of removing one expert data point at a time on abundance (N), detection probability (p) and misidentification (m). Expert data was available for 20 sites, the figures show the posteriors for each of the 20 model runs, which all overlap the model mean and posterior (shown in red)


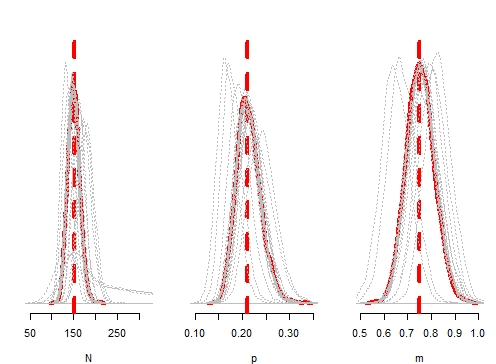


Figure S4. Validation analysis of IAM applied to expert data and survey data, illustrating the impact of removing one expert data point at a time on abundance (N), detection probability (p) and misidentification (m). Expert data was available for 21 sites, the figures show the posteriors for each of the 21 model runs, which all overlap the model mean and posterior (shown in red)

1. **Case study simulation**
   1. **Reports and expert count data**

*Methods*

To test the performance of the IAM on data comparable to that estimated from the case study we simulated pseudo-data to create 100 independent datasets that represented the field system. In accordance with the raw data, all 100 datasets simulated 22 sites, with variable numbers of citizen science counts per site that were identical to the raw data (range 1-42). Expert counts were available for 20 out of the 22 sites.

Further parameters were derived from the model outcomes, to test whether the IAM effectively performs under such scenarios. Consequently, three out of the 22 sites were randomly selected using a random number generator and simulated to be unoccupied. For a specified total population size of 143 cats, a multinomially distributed vector is computed as true site abundance for occupied sites. We used per site probabilities of cat abundance to simulate heterogeneity of cat abundance across sites. Thus, our simulated data sets did not assume similar probabilities of cat abundance across occupied sites.

The CS report data were simulated to have a detection probability of 0.14 and cat misidentification rate of three and additional variation via a Poisson distribution across all sites and count replicates.

Although expert counts are thought to be accurate in our case study, we do not have a direct measure of precision therefore we account for observation error within the IAM using a Poisson distribution. To test the effect of variability in expert counts we compare model runs where expert data were simulated to include observation error with model runs where expert data were simulated to be precise.

We looked at precision, accuracy and bias, according to definitions in main manuscript.

*Results*

The IAM performed without bias under scenarios simulated to represent the field system of the case study. All parameters had high accuracy (> 90%). The accuracy of abundance estimates and detection probability increased in scenarios where expert data were not simulated to include observation error. Thus, precision in expert estimates improved model accuracy for some parameters, but not for misidentification.


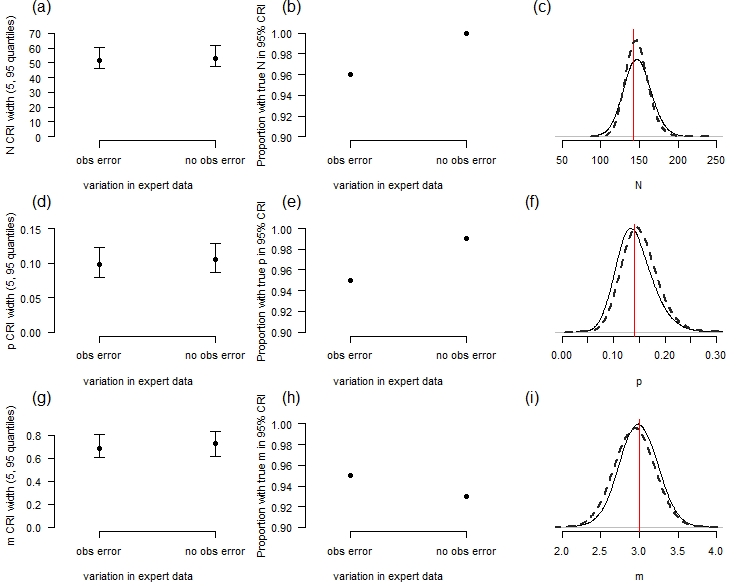


Figure S5. Precision(a,d,g), accuracy(b,e,h) and bias(c,f,I ) of abundance (N; a-c), detection probability (p; d-f) and misidentification (m; g-i) from models of data representative of the reports and expert data in the case study field system, in scenarios where expert data is simulated to include observation and to be precise. Precision is measured as the width of the credible intervals (CRI). Points and whiskers show the 50%, 5% and 95% quantiles, across replicate simulations, of the 95% CRI width for parameter estimates. Accuracy is measured here by the proportion of simulations where the true value is captured by the 95% CRI. Bias is observed as the full posteriors from all simulations: solid line = observation error in expert counts; dashed line=precise expert counts; red line=simulated values.

- 1. **Survey and expert count data**

*Methods*

To test the performance of the IAM on data comparable to that estimated from the case study we simulated pseudo-data to create 100 independent datasets that represented the field system. In accordance with the raw data, all 100 datasets simulated 28 sites, with variable numbers of citizen science counts per site (range 0-66). Expert counts were available for 21 out of the 28 sites.

Further parameters were derived from the model outcomes, to test whether the IAM effectively performs under such scenarios. Consequently, nine out of the 28 sites were randomly selected using a random number generator and simulated to be unoccupied. For a specified total population size of 151 cats, a multinomially distributed vector is computed as true site abundance for occupied sites. We used per site probabilities of cat abundance to simulate heterogeneity of cat abundance across sites. Thus, our simulated data sets did not assume similar cat abundance across occupied sites.

The CS survey data were simulated to have a detection probability of 0.21 and cat misidentification rate of one and additional variation via a Poisson distribution across all sites and count replicates.

Although expert counts are thought to be accurate in our case study, we do not have a direct measure of precision therefore we account for observation error within the IAM using a Poisson distribution. To test the effect of variability in expert counts we compare model runs where expert data were simulated to include observation error with model runs where expert data were simulated to be precise.

We looked at precision, accuracy and bias, according to definitions in main manuscript.

*Results*

The IAM performed without bias under scenarios simulated to represent the field system of the case study. All parameters had high accuracy (> 90%). The accuracy of abundance estimates and detection probability increased in scenarios where expert data were simulated to not include observation error. Thus, precision in expert estimates improved model inference for some parameters, but not for misidentification, where accuracy decreased.


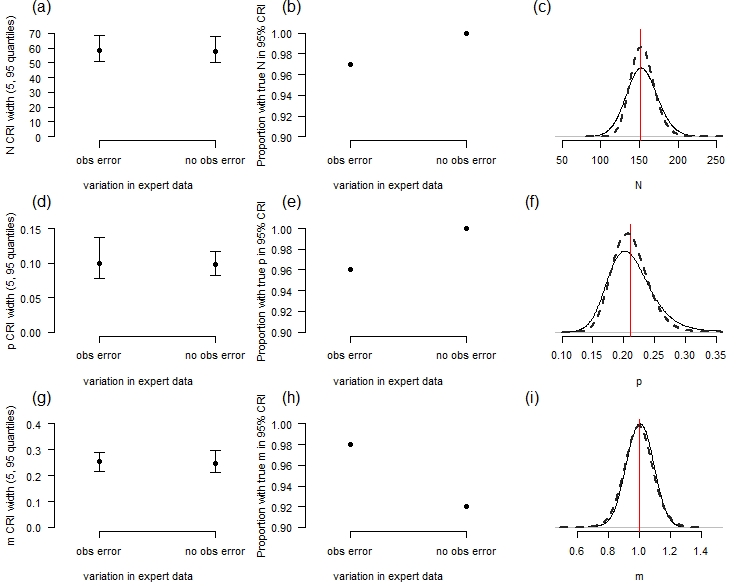


Figure S6. Figure S5. Precision(a,d,g), accuracy(b,e,h) and bias(c,f,I ) of abundance (N; a-c), detection probability (p; d-f) and misidentification (m; g-i) from models of data representative of the survey and expert data in the case study field system, in scenarios where expert data is simulated to include observation and to be precise. Precision is measured as the width of the credible intervals (CRI). Points and whiskers show the 50%, 5% and 95% quantiles, across replicate simulations, of the 95% CRI width for parameter estimates. Accuracy is measured here by the proportion of simulations where the true value is captured by the 95% CRI. Bias is observed as the full posteriors from all simulations: solid line = observation error in expert counts; dashed line=precise expert counts; red line=simulated values.
